# Barriers for Inter-Organisational Collaboration: What Matters for an Integrated Care Programme?

**DOI:** 10.5334/ijic.6005

**Published:** 2022-06-15

**Authors:** Angela Bångsbo, Anna Dunér, Synneve Dahlin-Ivanoff, Eva Lidén

**Affiliations:** 1 University of Gothenburg, Institute of Neuroscience and Physiology, and Centre of Health and Ageing, SE; 2 University of Gothenburg, Department of Social Work, and Centre of Health and Ageing, University of Gothenburg, SE; 3 University of Gothenburg, Institute of Health and Care Sciences, and Centre of Health and Ageing, SE

**Keywords:** integrated care, collaboration, inter-sectoral, continuity of patient care, personnel, frail older patients

## Abstract

**Introduction:**

Inter-organisational collaboration is challenging but essential in managing the complex and comprehensive needs of frail older people. Therefore, there is a need to investigate the influence of different barriers to inter-organisational collaboration when implementing an integrated care programme. The aim of this study was to investigate both inpatient and outpatient staff views on the factors they deemed to be influential to inter-organisational collaboration for an integrated care programme.

**Methods:**

The study was a cross-sectional study and included staff from hospitals, primary care and municipal health and social care.

**Results:**

There were no significant differences between staff from inpatient and outpatient care in measuring factors that may cause difficulties for inter-organisational collaboration. Staff views diverged significantly on all factors, such as educational level at long physical distances, laws and regulations, knowledge of each others work settings, experience from inter-organisational collaboration, different professions, variations in professional status and power, psychosocial factors such as positive work environment and interpersonal chemistry.

**Discussion:**

A multidisciplinary team culture and avenues for inter-organisational collaboration need to be developed for improved care continuity.

**Conclusion:**

The staffs’ educational level influenced what was perceived as barriers to inter-organisational collaboration, and may guide future development of integrated care programmes.

## Introduction

Integrated care programmes (ICPs) to coordinate and secure care continuity have been raised as essential to provide quality care of frail older people, and these programmes are recommended in the World Health Organization (WHO) guidelines as well as Swedish legislation [1,2,3,4]. Often, frail older people have complex and comprehensive needs, including both health (e.g. having multiple diagnoses) and social care (e.g. having financial hardships) that require care from various professionals across organisational boundaries [1]. Meeting the needs of and offering good quality integrated care to frail older people can thus be challenging to health and social care organisations. Thus, to ensure continuity of care from inpatient through outpatient care, ICPs should include collaboration, multi-professional teams and continuous staff education [5].

However, research has emphasised structural factors, such as a lack of mutual laws and policies and knowledge about each other’s working conditions [6,7], different administrative boundaries and information systems [8], which create barriers to inter-organisational collaboration. Moreover, there are factors related to the differences in commitments between the organisations, different interests and values and organisational and professional cultures that create barriers to collaboration between organisations. On an operational level, lack of experience of inter-organisational collaboration, communication deficits, the influence of different professions, professional status and power, psychosocial factors and diverging expectations of care responsibilities are also described as barriers to inter-organisational collaboration in the literature [9,10,11]. Furthermore, difficulties to inter-organisational collaboration have been emphasized between healthcare and social care staff [12]. Hence, there is a need to support [13] and to address the obstacles to inter-organisational collaboration when designing and implementing ICPs.

In Sweden, the municipalities have the overall responsibility for social care and services for older people, including special housing. However, health care is provided by both regions/county councils and municipalities, and the division of responsibility is more complex. Within the county councils the regional/local hospitals are responsible for acute medical care that requires hospital admission, and primary health care centers are responsible for outpatient care. Municipal healthcare is in charge of health care (except for medical care) for those living in special housing, and after local agreements for home nursing for those living in their own homes. In both health and social care, staff with as well as without academic degrees are found. Registered nurses, occupational therapists, physiotherapists, social workers and physicians all have at least a bachelor level university degree in their discipline, and thus regarded as academic professions. Whereas, practical nurses, including nurses’ aides, most often have a degree from upper secondary school and may be regarded as non-academic staff.

Both in Sweden and internationally, there is a lack of studies exploring how staffs’ organisational affiliation to inpatient or outpatient care and educational level may influence their views on various previously identified barriers to inter-organisational collaboration. Staffs’ educational levels and organisational affiliations may have an impact on how they interact in situations while collaborating with staff from other organisations. Moreover, the implementation of complex interventions depends on how the organisations and staff interact [14]. Challenges to implementing complex interventions are related to insufficient funding, knowledge and training among the staff [15]. Hence, external and internal factors, such as motivation and capability, may have an impact on implementation outcomes [16]. To create the best possible conditions for successful outcomes when implementing complex interventions, such as ICPs, staffs’ views are important and need to be integrated into the implementation process. Therefore, the aim of this study was to investigate the staffs’ views on which s barriers, measured as factors, that may impact inter-organisational collaboration in the context of health and social care for frail older people. We also aimed to compare the outcomes between inpatient and outpatient staff, and education level.

The current ICP was developed in collaboration with a university hospital, municipal health and social care, primary care staff and researchers at AgeCap, Centre for Aging and Health at the University of Gothenburg. The ICP includes comprehensive geriatric assessments in the emergency department followed by an assessment and discharge planning at home from a multi-professional team coordinated by a case manager. The purpose of the programme is to build an integrated continuum of care for frail older patients from the emergency unit through the hospital and back to community living. The programme was developed and tested in the Swedish context between 2012–2016 within the study “Continuum of Care for Frail Older People”, which designed a randomised, two-armed intervention study for frail older people who were living in the community. The intervention model was shown to improve the care quality of the frail older people compared to conventional care [17,18,19,20,21,22]. These promising results became the basis of a political decision to implement the model as a programme starting in 2012 [23,24]. Prior to the implementation, the staff performing the activities of the ICP, who all had an academic education, received training from the project leaders and researchers about the programme organisation, laws and guidelines that regulate the discharge process as well as the inter-organisational and professional collaborative approach to the programme. The other staff involved in performing health and social care for frail older people, both academic and non-academic staff mainly received information about the programme organisation.

## Methods

### Study design

This study was designed as a cross-sectional study to investigate the staffs’ views on inter-organisational collaboration within an ICP. The surveys were distributed in local units within hospital, primary care and municipal health and social care in an average sized municipality in Western Sweden. The survey was conducted in 2012.

### Question operationalisation and pilot testing

In this study, a questionnaire that was used in a similar study regarding the implementation and collaboration within child protection was modified and used [25]. The final questionnaire contained seven factors, which were based on previous research findings [6,7,8,9,10,11,12] that showed had an impact on inter-organisational collaboration (see Table 1). The modification comprised four steps, involving the care organisations, to be suitable for the health and social care context and the target group.

**Table 1:** Questions and abbreviations.


QUESTIONS	QUESTION ABBREVIATIONS

Assess the importance of the following factors for inter-organisational collaboration around frail older people. How do/does:	

– long physical distances between involved units influence collaboration?	Long distances

– different and possibly contradictory laws and regulations for different work and professional groups influence collaboration?	Laws and regulations

– insufficient knowledge about each other’s work settings and their particular conditions influence collaboration?	Knowledge

– insufficient experiences from inter-organisational collaboration influence collaboration?	Collaboration

– different types of professions influence collaboration?	Professions

– variations in professional status and power among the staff at the care units influence collaboration?	Status and power

– psychosocial factors (positive work environment, interpersonal chemistry e.g. openness to interdisciplinary work, communication skills) influence collaboration?	Psychosocial factors


The first step in the modification was based on the findings of a qualitative study of the implementation process of “Continuum of Care for Frail Older People” [24]. Second, the questionnaire was modified following the steering committee review. In the third step, staff at operative levels pilot tested the questionnaire. Finally, the questionnaire was revised and tested one last time in order to verify its validity [26].

The seven factors were chosen to investigate the staffs’ views on the barriers for inter-organisational collaboration within the ICP around frail older people. The factors were turned into questions to measure the staffs’ views on a visual analogue scale (VAS) ranging from 1 – no difficulty to 5 – great difficulties with a 0 - ‘don’t know’ alternative. The response alternatives were then categorised and dichotomised into no difficulty (response alternatives 0–3) and difficulties (response alternatives 4–5). The questions and their abbreviations are presented in Table 1.

### Data collection and participants

Initially, there was a multistage sampling among 32 units in a university hospital, primary care and municipal health and social care [27]. These units were then categorised as inpatient (hospital) and outpatient (primary and municipal health and social care). This multistage sample represented relevant care specialties at the hospital (geriatrics, orthopaedics, medicine, emergency department), health centres and responsibilities within the municipality (needs-assessment, home-care services, home nursing, rehabilitation). Thereafter, the units within each specialty or unit were proportionally randomised [27]. Twenty-four units from a university hospital, primary care and municipal health and social care were included. Six units dropped out initially, as a contract with a private home care provider had expired, one unit declined participation for organisational reasons, and four units were unreachable.

There were 18 units remaining, and the number of staff in each of the units varied between 8-45 staff. In inpatient care, physicians, occupational therapists and physiotherapists were organised at separate units at the hospital and were therefore excluded. Data collection took place at staff meetings within the units at the university hospital, health centres and municipal health and social care. All of these units were directly or indirectly involved with the ICP. There were various professional groups represented in the units with those with academic degrees like occupational therapists and physiotherapists, social workers, nurses and physicians. Non-academic staff were practical nurses including nurses’ aides, who were categorised as practical nurses. The total median age was 44 years, and the majority of the respondents were women and practical nurses. Descriptive statistics with demographic data are presented in Table 2.

**Table 2:** Demographic data of the staff in inpatient and outpatient care.


CHARACTERISTICS	INPATIENT CARE N = 48 (23%)	OUTPATIENT CARE N = 160 (77%)	TOTAL N = 208 (100%)

Median age (range)	43 (24–64)	45 (19–64)	44 (19–64)

Women, n (% valid)	44 (94)	142 (92)	186 (92)

Occupational therapists	0	15 (9)	15 (7)

Social workers	0	9 (6)	9 (4)

Physiotherapists	0	11 (7)	11 (5)

Nurses	27 (56)	23 (14)	50 (24)

Practical nurses	21 (44)	95 (59)	116 (56)

Physicians	0	7 (4)	7 (3)


### Procedure

Initially, the managers at the units were contacted by telephone and then by e-mail. They received oral and written information about the study, and they were asked to invite the researchers to a staff meeting. The researchers’ goals were to distribute and collect the questionnaires when the staff were gathered at the meetings and to attend the meetings to respond to any potential questions. Hence, at 12 out of 18 meetings, the questionnaires were distributed by the researchers. However, six managers returned the questionnaires by post since they distributed the questionnaires at a staff meeting themselves. Two people declined to participate.

### Data analysis

The results were summarised by frequencies and percentages, and Chi-square tests were carried out for group comparisons (i.e. inpatient versus outpatient care staff and academic versus non-academic staff) [27]. Statistical significance was set at p ≤ 0.05. Data analysis was performed using IBM SPSS Statistics for Windows, version 23.0 (IBM Corp., Armond, New York). Missing values were ≤ 4%.

### Ethics

This study was approved by the Swedish Research Ethical Committee, 2008, 2012 (Dnr 413–08 and T 140–12). The participants were given information in accordance to the research ethical standards based on the World Medical Association (WMA) Declaration of Helsinki’s [28] ethical principles for medical research involving human participants. The data were handled confidentially, and only the researchers had access to the responses.

## Results

There were 208 respondents in this study, representing both inpatient care (n = 48) and outpatient care (n = 160). A majority (n = 116) were non-academic and a minority (n = 92) had tertiary academic education. Having insufficient knowledge about each other’s work settings was the factor that the staff scored as associated with the greatest difficulty to inter-organisational collaboration when implementing an ICP (n = 132, 63%), as presented in Table 3. Having insufficient experience with inter-organisational collaboration was the second highest ranked factor that was perceived as creating difficulty for collaboration (n = 99, 48%), and psychosocial factors, such as insufficient work environments and interpersonal chemistry (n = 91, 44%) was third. The influence of different professions (n = 27, 13%) and professional status and power among the staff at the care units (n = 49, 24%) were the least indicated to cause collaborative difficulty. Furthermore, a large proportion of the staff did not know how laws and regulations (n = 95, 46%) or how status and power influenced inter-organisational collaboration (n = 77, 37%).

**Table 3:** Distribution (n or %) of factors to inter-organisational collaboration difficulties.


QUESTION ABBREVIATIONS	RESPONSE ALTERNATIVES TO DIFFICULTIES N = 208 (%)		

	A. YES	B. NO	C. DON’T KNOW

**Knowledge**	132 (63)	39 (19)	31 (15)

**Collaboration**	99 (48)	51 (25)	51 (25)

**Psychosocial factors**	91 (44)	47 (23)	62 (30)

**Laws and regulations**	65 (31)	40 (19)	95 (46)

**Long distances**	65 (31)	75 (35)	63 (30)

**Status and power**	49 (24)	75 (36)	77 (37)

**Professions**	27 (13)	111 (53)	62 (30)


Staff without an academic education responded that they did not know to a greater proportion of questions (24–56%) compared to staff with an academic education (4–37%). See Table 5.

### Comparing differences in views between inpatient and outpatient care staff

There were no statistically significant differences in the inpatient versus outpatient staff views when comparing potential difficulties to inter-organisational collaboration (see Table 4).

**Table 4:** Inpatient versus outpatient staff views of the factors that were considered difficulties to inter-organisational collaboration. 0–3 No difficulty, 4–5 Yes, difficulties; p-value Chi-square test.


QUESTION ABBREVIATIONS	RESPONSE ALTERNATIVES TO DIFFICULTIES	INPATIENT CARE N = 48 (%)	OUTPATIENT CARE N = 160 (%)	P-VALUE	TOTAL N = 208 (%)

Long distances	A. Yes	13 (28)	52 (34)	.124	65 (31)
	
	B. No	13 (28)	60 (39)		75 (35)
	
	C. Don’t know	20 (43)	43 (28)		63 (30)

Laws and regulations	A. Yes	12 (26)	53 (34)	.108	65 (31)
	
	B. No	6 (13)	34 (22)		40 (19)
	
	C. Don’t know	28 (61)	67 (44)		95 (46)

Knowledge	A. Yes	29 (63)	103 (66)	.653	132 (63)
	
	B. No	8 (17)	31 (20)		39 (19)
	
	C. Don’t know	9 (20)	22 (14)		31 (15)

Collaboration	A. Yes	21 (46)	78 (50)	.428	99 (48)
	
	B. No	10 (22)	41 (26)		51 (25)
	
	C. Don’t know	15 (33)	36 (23)		51 (25)

Professions	A. Yes	2 (4)	25 (16)	.127	27 (13)
	
	B. No	27 (60)	84 (54)		111 (53)
	
	C. Don’t know	16 (36)	46 (30)		62 (30)

Status and power	A. Yes	9 (20)	40 (26)	.089	49 (24)
	
	B. No	13 (28)	62 (40)		75 (36)
	
	C. Don’t know	24 (52)	53 (34)		77 (37)

Psychosocial factors	A. Yes	21 (46)	70 (45)	.442	91 (44)
	
	B. No	8 (17)	39 (25)		47 (23)
	
	C. Don’t know	17 (37)	45 (29)		62 (30)


### Diverging views between academic and non-academic staff

When comparing differences in views between staff with academic and non-academic educations, statistically significant differences occurred at all collaboration factors. See Table 5. Academic staff compared to the non-academic staff scored several factors higher as causing difficulty for inter-organisational collaboration: laws and regulations (p = .02), insufficient knowledge about each other’s work settings (p = .001) and insufficient experiences of inter-organisational collaboration (p = .002). On the other hand, non-academic staff scored other difficulties to inter-organisational collaboration higher: long distances (p = .02), different professions (p < .001), professional status and power among the staff at the care units (p = .02) and psychosocial factors, for instance insufficient work environments, interpersonal chemistry, (p = .01).

**Table 5:** Academic versus non-academic educated staff views of the factors that were considered difficulties to inter-organisational collaboration. 0–3 No difficulty, 4–5 Yes, difficulties; p-value Chi-square test.


QUESTION ABBREVIATIONS	RESPONSE ALTERNATIVES TO DIFFICULTIES	ACADEMIC EDUCATION N = 92 (%)	NON-ACADEMIC EDUCATION N = 116 (%)	P-VALUE	TOTAL N = 208(%)

Long distances	A. Yes	26 (29)	39 (35)	.02	65 (31)
	
	B. No	42 (47)	31 (28)		73 (35)
	
	C. Don’t know	22 (24)	41 (37)		63 (30)

Laws and regulations	A. Yes	36 (40)	29 (36)	.02	65 (31)
	
	B. No	21 (23)	19 (17)		40 (19)
	
	C. Don’t know	33 (37)	62 (56)		95 (46)

Knowledge	A. Yes	68 (76)	64 (57)	.001	132 (63)
	
	B. No	18 (20)	21 (19)		39 (19)
	
	C. Don’t know	4 (4)	27 (24)		31 (15)

Collaboration	A. Yes	52 (58)	47 (42)	.002	99 (48)
	
	B. No	25 (28)	26 (23)		51 (25)
	
	C. Don’t know	12 (13)	39 (35)		51 (25)

Professions	A. Yes	10 (11)	17 (15)	< .001	27 (13)
	
	B. No	65 (72)	46 (42)		111 (53)
	
	C. Don’t know	15 (17)	47 (43)		62 (30)

Status and power	A. Yes	20 (22)	29 (26)	.02	49 (24)
	
	B. No	43 (48)	32 (29)		75 (36)
	
	C. Don’t know	27 (30)	50 (45)		77 (37)

Psychosocial factors	A. Yes	40 (45)	51 (46)	.01	91 (44)
	
	B. No	29 (33)	18 (16)		47 (23)
	
	C. Don’t know	20 (22)	42 (38)		62 (30)


## Discussion

There were no significant differences between inpatient and outpatient care staff on factors that influenced inter-organisational collaboration. However, staff views diverged significantly on all factors when comparing academic to non-academic staff when implementing an ICP that requires inter-organisational collaboration.

The academic staff scored insufficient knowledge about each other’s work settings and their particular conditions as a difficulty to inter-organisational collaboration. This factor can be related to the lack of mutual awareness of each other’s work settings and their circumstances. However, previous studies have demonstrated the importance of staff education to overcome barriers, such as a lack of mutual knowledge of each other’s work settings, to inter-organisational collaboration [6]. Hence, this issue emphasises the importance of including knowledge about each other’s work settings and related conditions when planning the training that is involved in inter-organisational implementation.

The non-academic staff scored long physical distances between the involved units as a difficulty to inter-organisational collaboration. A possible explanation for this may be that the non-academic staff worked at premises that were far from the administrative buildings, hospital and health centre. Programme decisions are made to a high degree at the top management levels, presupposing top-down implementation of decisions where the non-academic staff, mainly from home-care services, had limited influence. Possibly, these management decisions are implemented without fully understanding the reality for the non-academic staff working conditions. Furthermore, a higher proportion of the non-academic staff selected the ‘don’t know’ response on all collaboration factors. A possible reason for this may be that the non-academic staff were practical nurses including nurses’ aides, and as such, not primarily responsible for collaboration and negotiation of care needs at patient discharge [29].

Studies have shown lower education, perceived lower status among social care workers, lack of knowledge and mistrust between staff from hospital and municipal health and social care were obstructing factors to collaboration [11]. Our findings revealed that lower education had a statistically significant influence on perceived difficulties to inter-organisational collaboration prior to implementing the ICP. According to Swedish law and the Higher Education Ordinance [30], obtaining a bachelor degree implies the graduate has a critical approach to their work and an ability to work independently. Hence, academically educated staff are of great benefit because academically educated nurses have been shown improve care quality and decrease patient mortality rates [31]. However, in our study, the majority of the non-academic respondents were workers from the municipal home care services, where there is a deficit in Sweden in relevant academic caring educations. Mistrust between staff from healthcare and social care was evident. Insufficient knowledge about each other’s work settings and their particular conditions was indicated as a difficulty that influenced collaboration within the ICP. Having preconceived ideas concerning other professions may serve as obstacles to collaboration among staff from healthcare and social care and have an impact on the development of care improvements [11,32,33,34].

To accomplish inter-organisational collaboration, multidisciplinary teamwork needs to meet the demand for specialisation and the need for care integration [35]. Teamwork is accomplished through shared decision-making, open communication and interdependent collaboration to improve patient, staff and organisational outcomes [36]. However, studies have shown that teamwork, contrary to its goals, strengthened professional hierarchies, occupational division and excluded groups of unlicensed staff from participating at work in healthcare [29,37]. Those results were evident in our study where lower educational levels among the staff significantly impacted collaboration. However, collaboration can be facilitated by good professional relationships, which can be implemented with training about each other’s professional roles even if they have not had previous experience with collaborative programmes [38]. Therefore, there is a need to develop avenues for inter-organisational collaboration for staff on the operative level to come together regardless of educational level.

To improve the preconditions for implementation of an ICP, a multidisciplinary team culture needs to be developed. Staff at the operative level should be included in the team to be able to be a part of building a mutual understanding of each other’s roles. Moreover, the development of new roles and competencies for integrated care has to be initiated by the management [39]. The results from the present study will form the basis of a longitudinal study between organisations of the implementation of an ICP from the staff’s understanding, commitment and ability to change their work procedures.

### Limitations

This study investigated the staffs’ views at one occasion—the start of the implementation of an ICP in a Swedish municipality. The units had heavy workloads with high employee turnover. This meant there were new staff with limited work experience, knowledge and experience of the inter-organisational collaboration programme. Hence, new staff may have selected the ‘don’t know’ response in the questionnaire. The researchers had control while collecting the questionnaires but not when the managers collected them, which may have influenced the responses. To respond to the aim and to not miss data, the factors were dichotomised into yes difficulties, and no difficulty including the ‘don’t know’ response. As occupational and physiotherapists in inpatient care were excluded, this caused a skewed sample towards nurses. The lack of significant differences between inpatient and outpatient staff may be caused by the uneven group sizes. Thus, a large sample size may have discovered differences. However, we estimate these results may be representative for all operative staff who were directly or indirectly involved in the ICP. Moreover, these limitations reflect ‘real-life’ conditions in many health and social care organisations. Our results could still increase our understanding of the influence different collaboration barriers have to complex organisational settings that are trying to implement more integrated and collaborative programmes between inpatient and outpatient care.

## Conclusion

Care of frail older people is often fragmented, including lack of integration and continuity between involved care organisations as well as between staff at different levels, and result in failures to meet their complex needs. Developing and implementing ICPs, to improve inter-organisational collaboration in this context, may overbridge these deficiencies. However, it is important to identify and address the barriers to integrated care that involved staff view as most obstrusive. In doing so diverging views according to organisational affilliation and/or educational level has to be taken into consideration. In this study, we show that educational level influence the views on barriers to inter-organisational collaboration, results that may guide future development and implementation of ICPs.
